# Functions and mechanisms of non-histone post-translational modifications in cancer progression

**DOI:** 10.1038/s41420-025-02410-2

**Published:** 2025-03-31

**Authors:** Zongyang Li, Tao Zhu, Yushu Wu, Yongbo Yu, Yunjiang Zang, Lebo Yu, Zhilei Zhang

**Affiliations:** 1 Department of Urology, The First Affiliated Hospital of Shandong Second Medical University, Weifang, 261041 China; 2 School of Clinical Medicine, Shandong Second Medical University, Weifang, 261041 China

**Keywords:** Epigenetics, Cancer epigenetics

## Abstract

Protein post-translational modifications (PTMs) refer to covalent and enzymatic alterations to folded or nascent proteins during or after protein biosynthesis to alter the properties and functions of proteins. PTMs are modified in a variety of types and affect almost all aspects of cell biology. PTMs have been reported to be involved in cancer progression by influencing multiple signaling pathways. The mechanism of action of histone PTMs in cancer has been extensively studied. Notably, evidence is mounting that PTMs of non-histone proteins also play a vital role in cancer progression. In this review, we provide a systematic description of main non-histone PTMs associated with cancer progression, including acetylation, lactylation, methylation, ubiquitination, phosphorylation, and SUMOylation, based on recent studies.

## Facts

- Epigenetic modifications can induce carcinogenesis and progression by influencing metabolic reprogramming. PTMs have become an important area of cancer research.
- Histone PTMs regulate the initiation, development, and treatment of cancer through a variety of signaling pathways.
- Targeting PTMs-related pathways is expected to be an effective means of cancer therapy.

## Open questions

- Whether non-histone PTMs also play an important role in cancer progression?
- What are the non-histone substrates for common PTMs and what are the mechanisms by which they regulate cancer progression?
- How different types of PTMs regulate cancer progression by forming regulatory networks?

## Introduction

Epigenetic remodeling is one of the characteristics of cancer [1]. Aberrant regulation of epigenetic modifications can induce tumorigenesis and tumor progression through metabolic reprogramming of cancer cells [2, 3]. Tumor-targeted therapies based on epigenetic modifications also provide us with new promising options. There is a complex interaction between cancer epigenetics and cancer immunology [4]. Epigenetic modifications can drive immune evasion or hinder immune surveillance, playing an important role in tumor progression [1].

Post-translational modifications (PTMs) are a means of altering the properties and functions of proteins after biosynthesis. Various PTMs, such as acetylation, lactylation, ubiquitination, methylation, and phosphorylation, have been reported to play a role in the progression of cancer [5–7]. The study of PTM has become an important area of cancer research, and most of the studies were conducted on histone proteins [8]. However, accumulating studies revealed that PTMs of non-histone proteins also play a non-negligible role in cancer [9, 10].

In this review, we discuss the functional impact of epigenetic changes in non-histone proteins, and how they contribute to tumor progression and their relevance to epigenetic treatments.

## Acetylation

Since the first non-histone protein p53 was found to be regulated by acetylation in the 1980s, thousands of non-histone proteins have been identified as acylation targets. Lysine acetylation is a widespread and versatile protein PTM. Like histones, non-histone proteins are often acetylated and form a major part of the acetylome in mammalian cells [11]. Lysine acetyltransferases (KATs) and lysine deacetylases (KDACs) catalyze the addition and removal of acetyl groups at histone and non-histone targets. In the enzymatic dependent condition, the acyltransferase—“writer” is responsible to add acyl groups from the “donors” of acyl-CoA to the side-chain of lysine residues. The deacylase—“eraser” catalyzes the removal of acyl groups from the aforementioned amino acid residues. The specific protein domains—“reader” read the acylation marks [12]. KDACs fall into two subgroups: Zn^2+^-dependent histone deacetylases (HDACs) and nicotinamide adenine dinucleotide (NAD^+^)-dependent sirtuins. Proteome-wide surveys have uncovered acetylation of thousands of non-histone proteins in the cell, including proteins involved in metabolism, RNA processing, translation, protein folding, chromatin organization, protein degradation, and cytoskeleton organization [13, 14]. Possible means by which non-histone acetylation may modulate protein function include altering its conformation, stability, hydrophobicity or localization, or blocking its capacity to accept other post-translational protein modifications [15, 16].

**Fig. 1:**
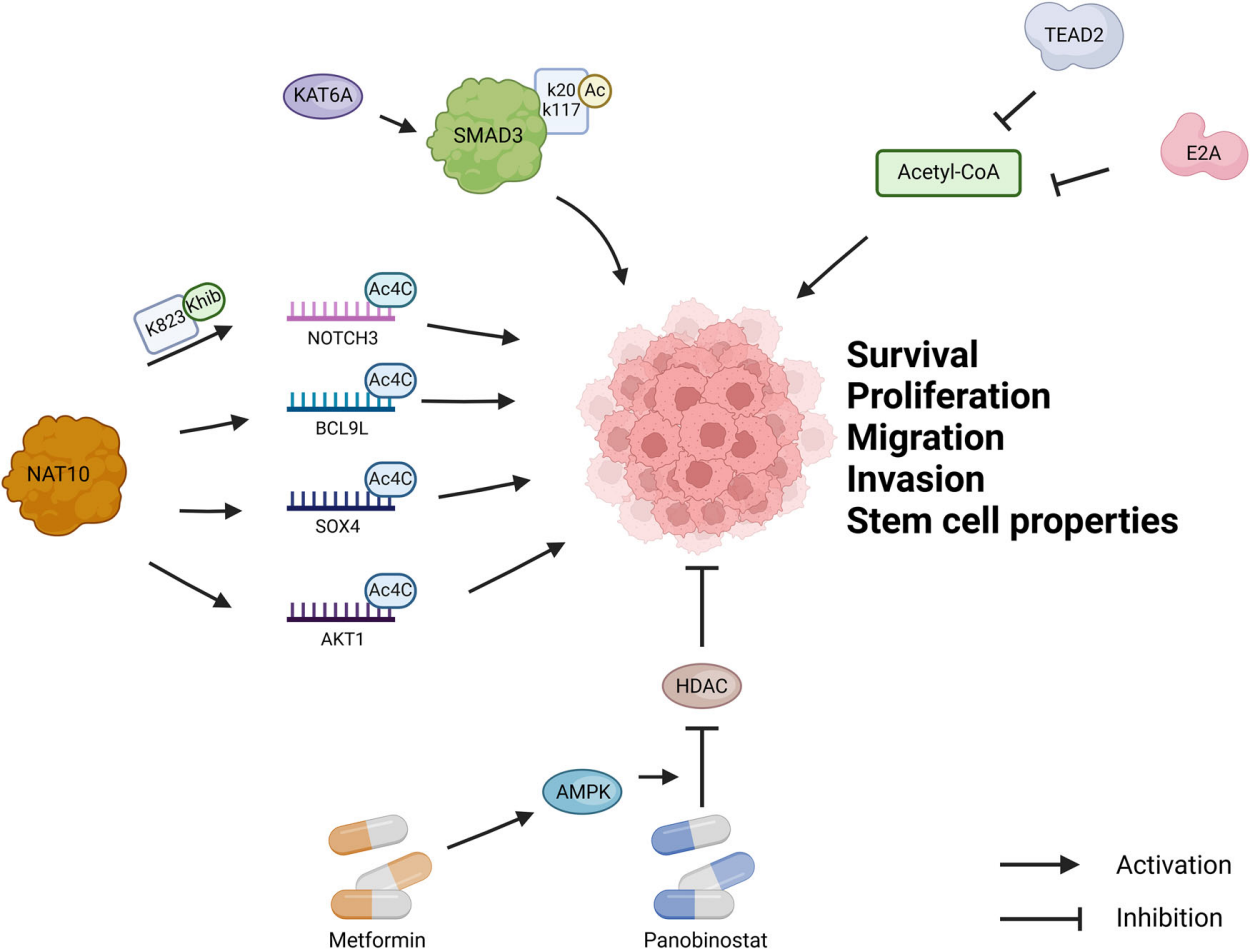
Non-histone acetylation modifications regulate cancer progression.

Protein acetylation mediated by KATs can occur in either tumor cells or immune cells and eventually change the intrinsic tumor features or immune cell phenotypes. Vigorous glycolysis in tumors is also closely related to acetylation. Next, we highlight several examples of non-histone acetylation modifications regulating cancer progression (Fig. 1).

EP300 encodes a lysine acetyltransferase that regulates a large subset of genes by acetylating histones and non-histone proteins. In bladder cancer, EP300 mutation was associated with higher tumor mutational burden scores and indicated a favorable clinical prognosis, which was reported to be a biomarker for immunotherapy [17]. According to the former study, combined CREB-binding protein and p300 inhibition impaired bladder cancer cell proliferation and induced apoptosis by decreasing c-Myc expression [18]. By screening the mutations of EP300, the EP300-R1627W mutation was found that significantly impairs EP300 transactivation activity following a “gradation” pattern, which promotes the growth and invasion of bladder cancer [19].

As a member of the SMAD proteins family, SMAD3 acts as a mediator of the TGF-superfamily to regulate signaling, regulating cell proliferation, apoptosis, immune monitoring, and cancer metastasis [20]. Aberrant SMAD3 activation is thought to be a driving event for cancer metastasis, but the underlying mechanism remains elusive. SMAD3 was identified as a non-histone substrate of lysine acetyltransferase 6 A (KAT6A) and the acetylation of SMAD3 at K20 and K117 by KAT6A leads to enhanced breast cancer stem-like cell stemness, myeloid-derived suppressor cell recruitment, and triple-negative breast cancer (TNBC) metastasis [21].

N4-acetylcytidine (ac4C) is a mRNA modification catalyzed by the N-acetyltransferase 10 (NAT10) [22]. By comparing a panel of non-histone lysine acylation patterns in metastasis models and clinical samples, Liao et al. [23] identified NAT10 as a non-histone substrate for lysine 2-hydroxyisobutyrylation (Khib) at lysine 823 site. This Khib modification stabilizes NAT10 protein by enhancing its interaction with deubiquitinase USP39 and then NAT10 increases NOTCH3 mRNA stability in ac4C manner, functionally contribute to cancer metastasis. Besides, NAT10 is highly expressed in BLCA patients and its unusual level predicts bladder cancer progression and low overall survival rate. NAT10 was responsible for ac4C modification of target transcripts in BLCA cells, including BCL9L, SOX4, and AKT1 and played an important role in the proliferation, migration, invasion, survival, and the stem-cell-like properties of BLCA cell lines [24].

Acetyl-CoA plays an important role in metabolism, gene expression, signaling, and other cellular processes through the transfer of its acetyl group to proteins and metabolites. Park et al. [25] found that acetyl-CoA levels are decreased in hepatocellular carcinoma due to transcriptional downregulation of all six acetyl-CoA biosynthesis pathways, which reduced acetylation levels of non-histone proteins and then promoted oncogenic dedifferentiation and proliferation. Mechanistically, acetyl-CoA synthesis is inhibited by the transcription factors TEAD2 and E2A [25].

In addition to acetylation, aberrant deacetylation of non-histone proteins controlled by HDACs is implicated in cancer progression as well. HDAC inhibitors (HDACi) regulate gene expression by restoring acetylation levels. HDACi have emerged as a novel and promising cancer treatment [26]. For example, Panobinostat has used as anticancer drug. Notably, combination with metformin can enhance its curative effect [27, 28]. Because the AMPK activation by metformin enhanced panobinostat-induced histone and non-histone acetylation [28].

## Lactylation

Lysine lactylation (Kla), is a form of PTM that involves the addition of a lactyl group (a molecule derived from lactic acid) to a lysine residue in proteins. This novel modification was first reported in 2019 [29], and has since been identified as a widespread PTM, affecting both histone and non-histone proteins. Lactylation of non-histone proteins is emerging as an important regulatory mechanism in various cellular processes. Unlike histone proteins, which are primarily involved in chromatin structure and gene regulation, non-histone proteins encompass a wide range of functional proteins that participate in virtually all cellular activities [30]. The lactylation of these proteins can have far-reaching effects on cellular function. For example, lactylation has been implicated in the regulation of metabolic pathways, inflammation, and immune responses. The lactyl group can be added to lysine residues by lactate dehydrogenase (LDH) or other enzymes, and its removal is thought to be mediated by lactyllysine-specific proteases or other lactyl-removing enzymes.

Even under completely aerobic conditions, cancer cells produce lactic acid and adenosine triphosphate (ATP) through glycolysis, a phenomenon known as the Warburg effect. Increased lactate production leads to acidification of the tumor microenvironment (TME), which favors tumor cell growth and survival [31]. Excess lactate helps to establish an immunosuppressive environment conducive to the growth of cancer cells and plays an important role in the function of immune cells [32]. Studies have confirmed that protein lactation can significantly affect its function and subsequent biological behavior of cancer cells [29, 30]. What’s more, Kla in non-histone proteins could be a prognostic marker in cancer patients [33].

Alanyl-tRNA synthetase 1 (AARS1), which has been discovered to function as a lactyltransferase, catalyzes the process of protein lactylation by utilizing lactate and ATP. This enzyme is capable of sensing the levels of intracellular lactate and subsequently relocating to the nucleus, where it directly lactylates YAP at K90 and TEAD1 at K108, thereby activating downstream target gene expression to promote tumor cell proliferation [34]. Besides, AARS1 has also been identified as a gene that is regulated by the Hippo pathway and, in turn, forms a self-reinforcing positive feedback loop with the YAP-TEAD1 complex to enhance the proliferation of gastric cancer cells [34]. Proteomic studies have revealed a large number of AARS1 targets, including p53. Lactylation of p53 at lysine 120 and lysine 139 in the DNA binding domain hinders liquid-liquid phase separation, DNA binding, and transcriptional activation, which contributes to tumorigenesis [35]. AARS1 expression and p53 lacylation correlate with poor prognosis among cancer patients [35, 36].

Sirtuin 3 (SIRT3) is a member of the Sirtuins family of NAD^+^-dependent proteins lysine decarboxylases [37]. SIRT3 has been implicated in a variety of cancers [38–40]. Deletion of SIRT3 triggers metabolic reprogramming (known as the Warburg effect), which promotes tumorigenesis [41, 42]. Importantly, SIRT3 has delactylation activity [43], the removal of lactylation from the non-histone proteins on Cyclin E2 (CCNE2), a key cell cycle protein reported to be essential for cancer progression [44–46], is catalyzed by SIRT3 to suppress the proliferation, migration, and invasion of cancer cells [47].

Copper is an essential trace element in living organisms and is involved in the regulation of cell growth and metabolism [48–50]. Copper content is markedly elevated in malignancy, and elevated serum copper levels in cancer patients are associated with cancer progression and chemotherapy resistance [51, 52]. Copper promotes angiogenesis by activating several pro-angiogenic factors, namely vascular endothelial growth factor (VEGF), fibroblast growth factor 2 (FGF2), tumor necrosis factor (TNF), and interleukin-1 (IL-1), thereby promoting tumor initiation, growth, and metastasis [53]. In addition, copper regulates tumor cell autophagy by affecting the activity of the autophagy kinases ULK1 and ULK2 (ULK1/2) [54]. However, if the concentration of copper exceeds a certain threshold, it becomes toxic, triggering a distinct form of cell death called cuproptosis [55]. FDX1 and protein lipacylation are key factors in the regulation of cuproptosis. FDX1 is a reductase known to reduce Cu^2+^ to a more toxic form Cu^1+^ [56]. Besides, FDX1 is an upstream regulator of protein lipacylation. The aggregation and loss of function of lipacylated proteins lead to the instability of iron–sulfur cluster proteins, leading to protein toxic stress and ultimately cell death [55]. High copper content promotes lactation of non-histone METTL16 at K229 by increasing the interaction of the potential lactate transferase AARS1/AARS2 with METTL16, and then lactated METTL16 upregulates FDX1 expression level by m6a modification of FDX1 mRNA, which ultimately leads to cuproptosis [57]. This suggests that METTL16 is the central hub that coordinates lactylation and copper metabolism in cancer.

**Fig. 2:**
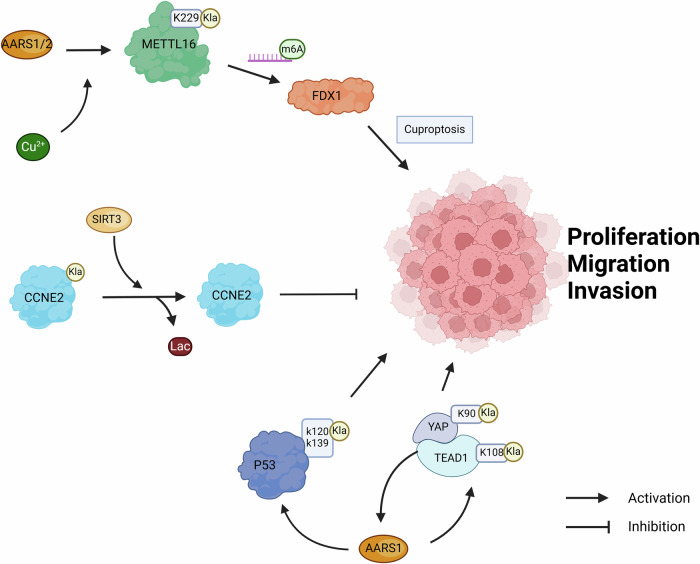
Non-histone lactylation modifications regulate cancer progression.

Sum up, there is reason to believe that Kla is an important hub connecting lactate, tumor metabolism and patient prognosis (Fig. 2). The mechanism of action of non-histone lactation in cancer deserves more in-depth study. Treatments targeting non-histone lactated targets are expected to be an effective treatment for cancer.

## Methylation

Lysine methylation, catalyzed by protein lysine methyltransferases (PKMTs), is one of PTMs implicated in various biological functions. Previously, the focus of methylation research has mostly shed light on the methylation of histones, and less on the methylation of non-histone proteins. However, methylation of non-histone proteins also plays an important role in human cancer [58, 59].

The protein arginine methyltransferases (PRMTs) family is an important group of epigenetic targets responsible for catalyzing the methylation of various intracellular substrates [60]. PRMT6 belongs to the family with the ability to methylate histones and non-histones [61]. PRMT6 plays different roles in various cancers by influencing cell growth, migration, invasion, apoptosis, and drug resistance [62]. Such as, PRMT6 asymmetrically di-methylates STAT3 at arginine-729 (R729) and this modification is essential for membrane localization of STAT3, interaction with JAK2, phosphorylation of STAT3 Y705, and PRMT6-driven cancer cell metastasis [63]. Besides, methylation of PRMT6 at RCC1 R214 regulates proliferation, stem-like properties, and tumorigenicity of glioblastoma stem cells by CK2α-PRMT6-RCC1 signaling axis [64]. Notably, there is growing evidence that non-histone methylation catalyzed by PRMT6 has a profound impact on tumor initiation and progression [65, 66]. Similarly, PRMT5 is highly over-expressed in a variety of aggressive metastatic cancers and dysregulation of its activity has been implicated in the development and progression of cancers [67–69]. Multiple mechanisms about methylation are involved. For example, PRMT5 regulates the activation of protein kinase B (AKT) through direct methylation of arginine-15 (R15), and AKT signaling controls the expression of the epithelial–mesenchymal-transition (EMT) transcription factors ZEB1, SNAIL, and TWIST1, which coordinates the EMT procedures responsible for tumor metastasis [70]. In addition, PRMT5 contributes to the inactivation of the Hippo signaling axis, a tumor suppressor pathway [71]. PRMT5 symmetrically di-methylates the Hippo pathway initiator serine/threonine kinase 3 (STK3, or MST2) at arginine-461 (R461) and arginine-467 (R467). Then, methylation inhibits STK3 autophosphorylation and kinase activity by blocking the homodimerization of STK3, thereby inactivating the Hippo signaling pathway [71]. In conclusion, the aberrant expression of PRMT is closely related to the occurrence, deterioration, metastasis, and drug resistance of tumors. Based on this, several studies have shown that PRMT inhibitors can be an effective means of fighting tumors [72–76].

Smad nuclear-interacting protein 1 (SNIP1) is a non-histone substrate of the lysine methyltransferase KMT5A. KMT5A-mediated SNIP1 K301 methylation was identified as a crucial initiation event for subsequent c-MYC activating the key oncogenic pathway-Hippo signaling, which is not only a key signal of cancer metastasis, but also a marker of poor prognosis in TNBC patients [77].

**Fig. 3:**
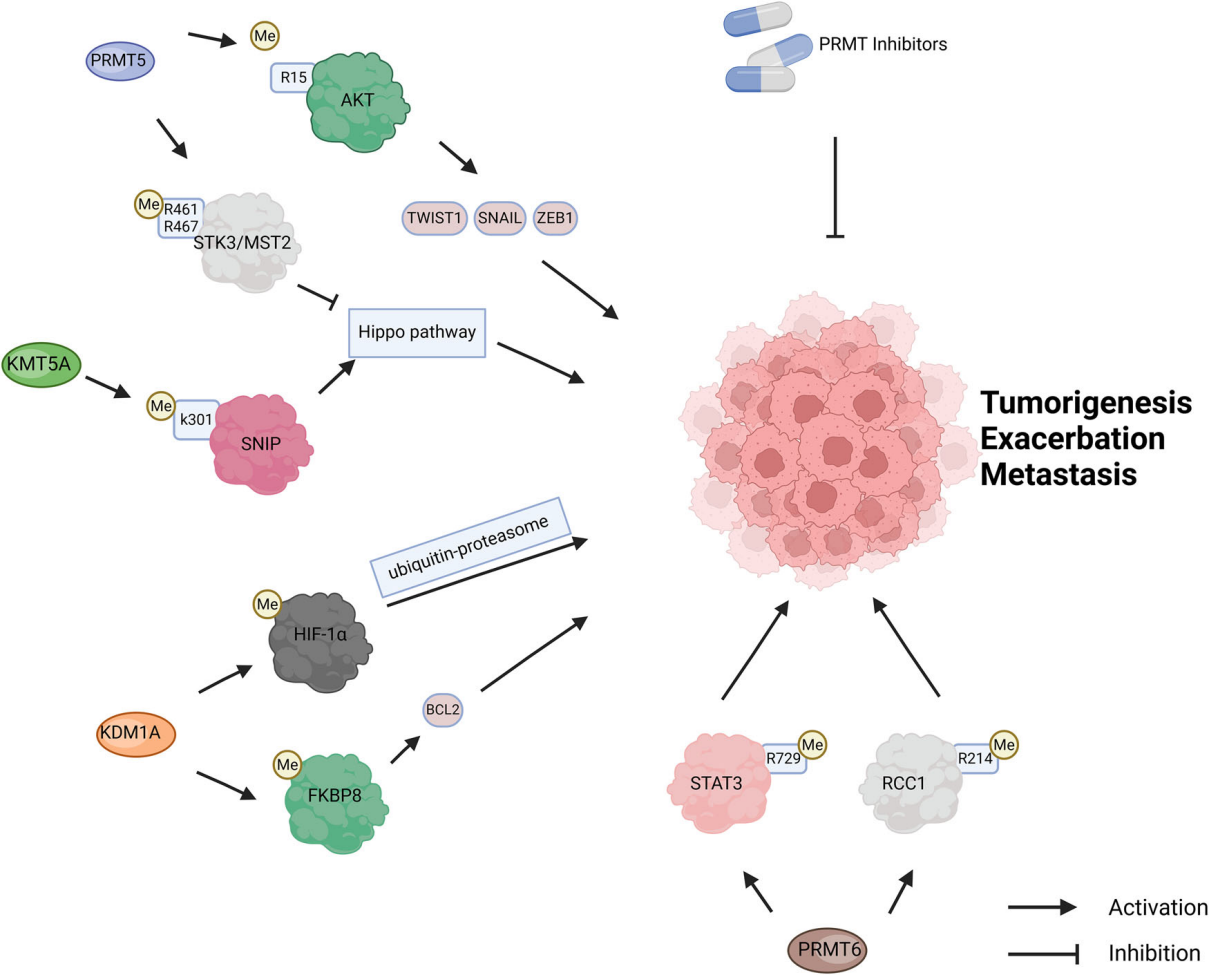
Non-histone methylation modifications regulate cancer progression.

Therefore, non-histone substrates of this class of histone methylation modifiers are of concern, and the role of non-histone proteins in these mechanisms warrants more in-depth study. Besides, demethylating modifications of non-histone proteins have also been involved in tumor progression (Fig. 3).

Lysine-specific histone demethylase 1 A (KDM1A, also known as LSD1) was the first histone lysine demethylase to be discovered [78]. However, recent studies have shown that KDM1A can also demethylate non-histone substrates [79]. For example, KDM1A can directly demethylate HIF-1α, a non-histone protein, and prevent its degradation through the ubiquitin-proteasome pathway. HIF-1α further regulates the stemness of thyroid cancer and promotes cancer progression via the HIF-1α/microRNA-146a/DKK1 axis [80]. FKBP8 is a member of the FK506-binding protein (FKBP) family and regulates cell survival through the anti-apoptotic protein B-cell lymphoma-2 (BCL2). KDM1A can interact with and demethylate FKBP8, enhancing its ability to stabilize BCL2, which promotes cancer cell growth and the development of acquired drug resistance [81]. At present, a number of studies on KDM1A inhibitors in the treatment of malignant tumors have made progress [82–84]. This effect can be attributed to a significant reduction in MYC signaling, and MYC was found to be a consistent target of KDM1A [85]. In addition, KDM1A formed a network with BRD4 and FOXA1 and was enriched in the super-enhancer region, exhibiting liquid–liquid phase separation [85]. Notably, proteomic analysis studies have shown that BRD4 can recognize non-histone proteins containing Kac-XX-Kac motif [86].

## Ubiquitination

**Fig. 4:**
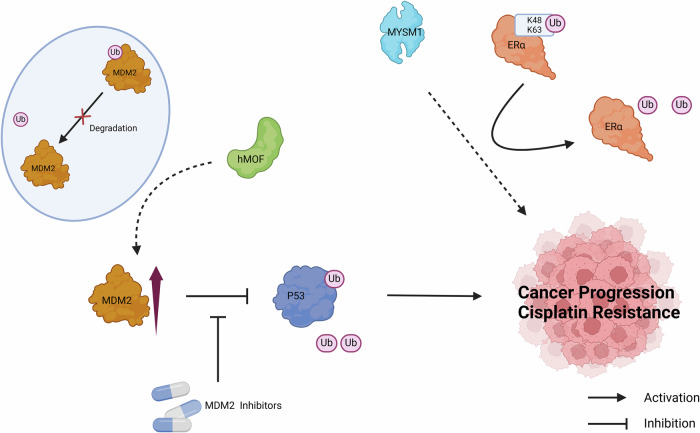
Non-histone ubiquitination modifications regulate cancer progression.

Ubiquitination is an important post-translational modification that regulates the localization and stability of substrate proteins, including histones and non-histones [87]. Ubiquitination is the result of the sequential interaction of E1-activating enzymes, E2-conjugating enzymes, and E3 ligases, resulting in ubiquitin covalently conjugated to lysine residues on proteins or on itself [88]. Simultaneously, deubiquitinating enzymes are responsible for removing these ubiquitin labels. Ubiquitination is ubiquitous, and it plays a significant role in maintaining genome stability and regulating most nuclear signaling pathways [89]. Previous reports have shown that histone ubiquitination regulates a variety of DNA-driven processes, and its aberrant expression often occurs in cancer [90–92]. Notably, accumulating evidence suggests that non-histone ubiquitination is also associated with various human cancers by regulating cell cycle, cell proliferation, DNA repair, apoptosis, inflammation, immune response, and so forth [93–95]. Therefore, it is of great significance to study the mechanism of non-histone ubiquitination and deubiquitination in tumor progression and treatment (Fig. 4).

Human males absent on the first (hMOF) is a histone acetyltransferase involved in the pathogenesis of various cancers [96, 97]. However, the substrate of hMOF is not only histones, but also non-histones. For example, p53, a critical tumor-suppressor gene exerting anticancer function by inducing apoptosis, is a non-histone substrate of hMOF [98, 99]. Murine double minute 2 (MDM2) is a key negative regulator of p53, induces the ubiquitination and degradation of p53 [100]. Accordingly, MDM2 inhibitors targeting the interaction between p53 and MDM2 have emerged as a promising therapeutic strategy [101, 102]. Additionally, the E3 ligase MDM2 protein is a novel non-histone substrate for hMOF. hMOF can directly interact with MDM2 and promote the accumulation of MDM2 by inhibiting its ubiquitination degradation, thereby inducing cisplatin resistance in tumor cells [103].

Endocrine resistance is a key challenge in estrogen receptor α (ERα)-positive breast cancer (BCa) [104]. A series of ER cofactors are involved in estrogen-driven transcriptional programs, providing selective advantages for cancer progression and endocrine resistance [105, 106]. Myb-Like SWIRM and MPN domains 1 (MYSM1) is a chromatin-binding protein with deubiquitinase catalytic activity. Previous studies have shown that MYSM1 is involved in the pathological process of tumors [107, 108]. But they focused on the role of only histone ubiquitination in cancer mechanisms. Luan et al. [109] suggest that MYSM1, in addition to its epigenetic regulatory function as an ERα coactivator, is also involved in non-histone modifications to maintain the stability of ERα in the progression of ERα-positive BCa. Mechanically, MYSM1 is involved in maintaining ERα stability by reducing lysine 48 (K48) and K63-linked poly-ubiquitination on ERα through non-histone deubiquitination to promote tumor cell proliferation and anti-estrogen insensitivity [109].

## Phosphorylation

**Fig. 5:**
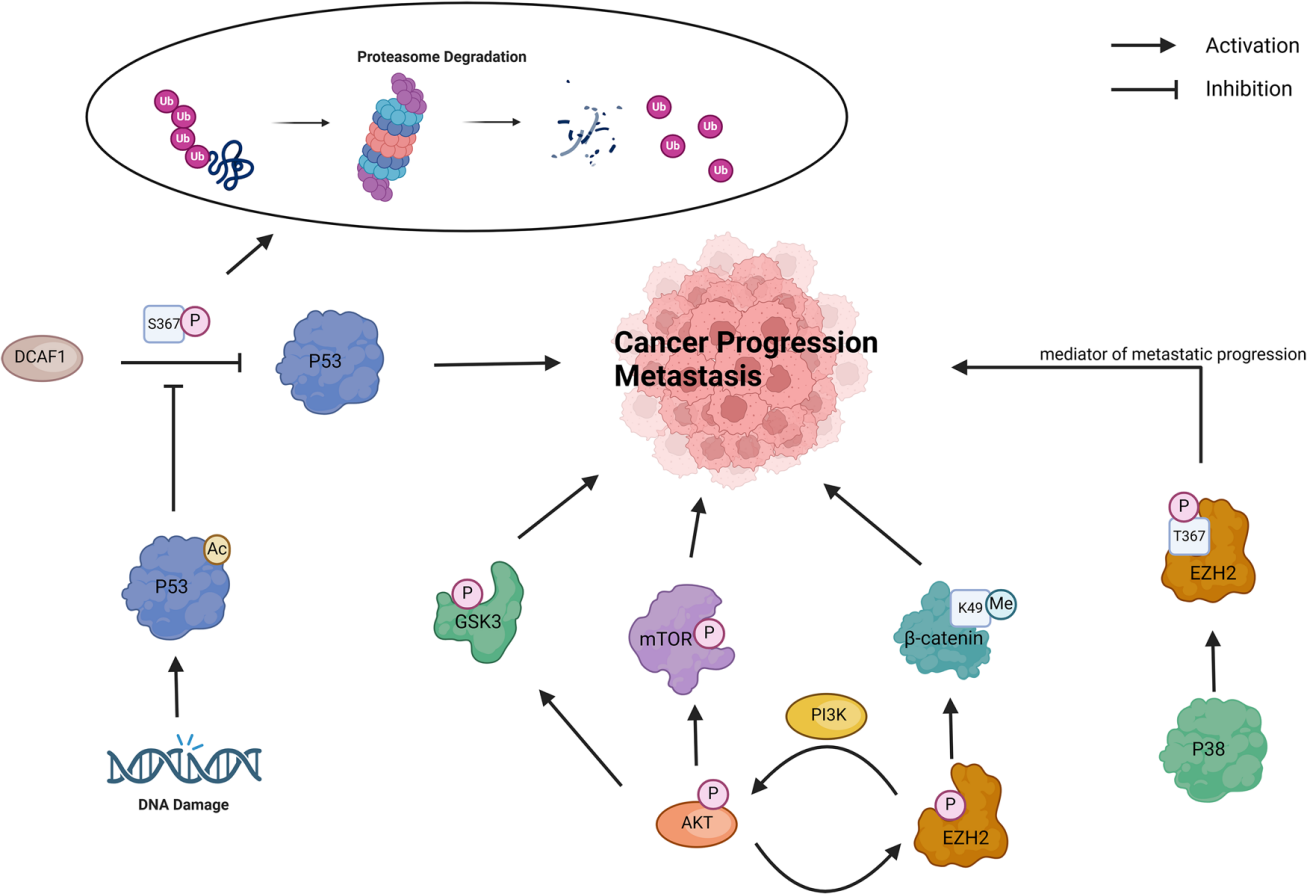
Non-histone phosphorylation modifications regulate cancer progression.

Phosphorylation is one of the most widely studied PTMs and its dysregulation usually leads to a disorder of cell proliferation, leading to tumorigenesis and tumor progression. Following, the latest mechanisms by which non-histone phosphorylation modifications regulate cancer progression will be described (Fig. 5).

The DDB1 and CUL4 associated factor 1 (DCAF1), also known as VprBP (HIV-1 Vpr binding protein), is a regulator of cell cycle and cell proliferation. DCAF1 is expressed at elevated levels in a variety of cancers and promotes cancer progression and metastasis through multiple signaling pathways [110–112]. DCAF1 inactivates growth regulatory genes in an H2AT120 phosphorylation (H2AT120p)-dependent manner, as point mutations in t120 in H2A eliminate the ability of DCAF1 to inhibit transcription against chromatin background [113]. Most studies have focused on the histone modification function of DCAF1 as a CUL4A E3 ubiquitin ligase complex. Notably, many histone-modifying enzymes also target non-histone proteins. DCAF1 catalyzes serine 367 phosphorylation (S367p) by direct interaction with the c-terminal domain of p53 and this site-specific phosphorylation inhibits p53 function by promoting proteasomal degradation [114]. Moreover, acetylation of p53 in response to DNA damage inhibits DCAF1 kinase activity towards p53 S367 by blocking the binding of DCAF1 to p53 and increasing the stability of p53, thereby enhancing the expression of target genes [114].

Enhancer of zeste homolog 2 (EZH2) is a highly conserved histone lysine methyltransferase that usually catalyzes the trimethylation of nucleosome histone H3 at lysine 27 (H3K27me3). EZH2-mediated H3K27me3 initiates tumorigenesis and inhibits the expression of tumor suppressor genes through multiple mechanisms [115–117]. Furthermore, EZH2 also has many non-histone substrates. For example, phosphorylation of EZH2 by AKT induced EZH2 to interact with and methylate b-catenin at lysine 49, thereby increasing the binding of β-catenin to chromatin [118]. In addition to AKT/EZH2/β-catenin axis, EZH2 also can promote the progression of cancer through the activation of the AKT/GSK3, PI3K/AKT/mTOR, and other pathways [119–121]. A recent study has demonstrated that DCAF1 phosphorylates the T367 site of EZH2 to enhance its nuclear stability and enzymatic activity in colon cancer cells, resulting in elevated H3K27me3 levels and altered growth-regulating gene expression in cancer cells [122]. Besides, EZH2 phosphorylation at T367 mediated by p38 potentiates EZH2 cytoplasmic localization and its role as a mediator of metastatic progression of breast cancer [123]. These results suggest that histone modification and non-histone modification can couple to regulate gene transcription. This crosstalk between multiple different kinds of PTMs can integrate various signals and greatly improve their regulatory capabilities.

## Sumoylation

**Fig. 6:**
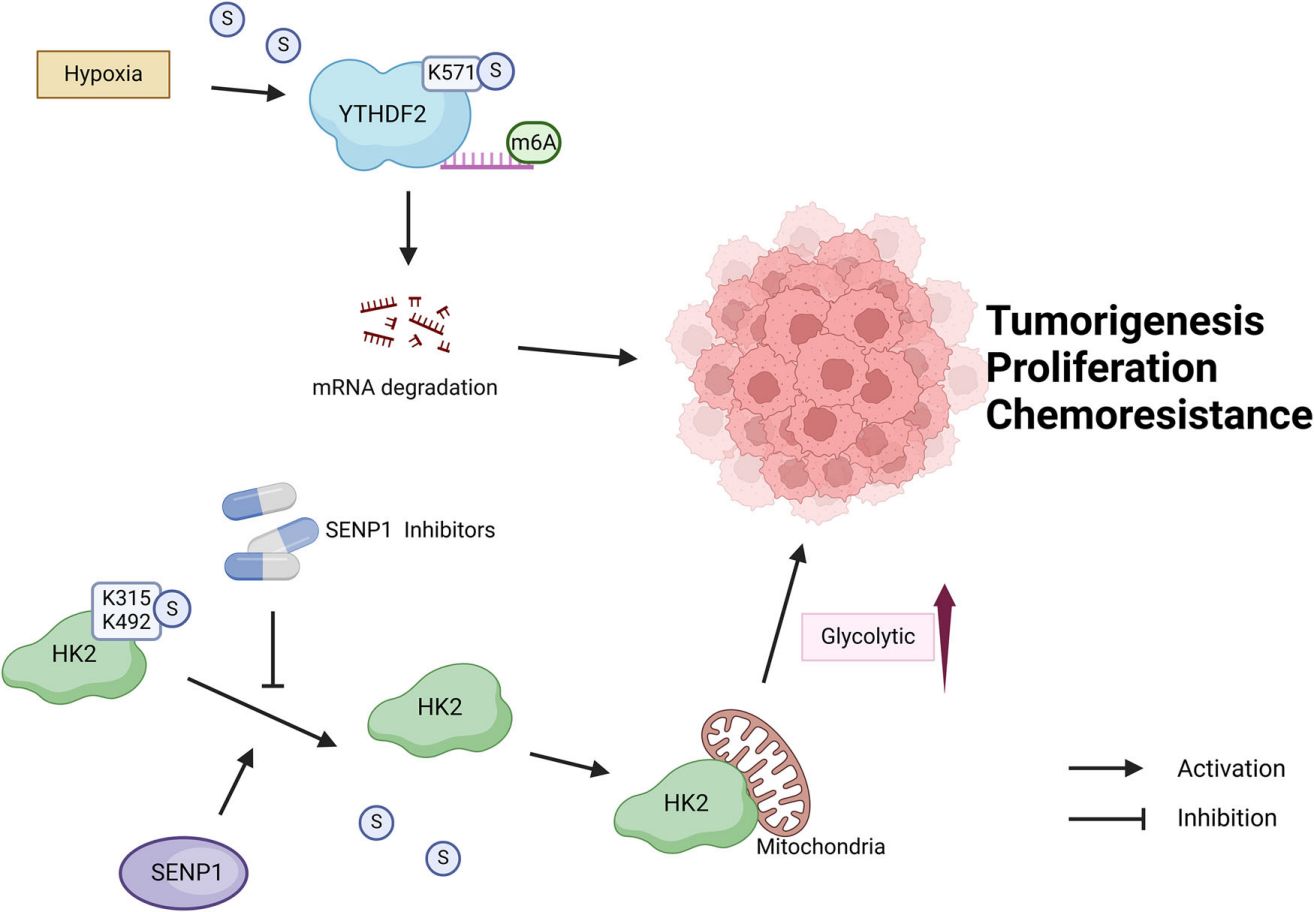
Non-histone SUMOylation modifications regulate cancer progression.

Small ubiquitin-like modifier (SUMO) is one of several proteins that have been found to be similar to ubiquitin in recent years. SUMO modifies the target protein by forming covalent bonds with specific lysine branched chains on the target protein through a process similar to ubiquitination, known as SUMOylation [124]. SUMO-specific proteases (SENPs) can not only catalyze the maturation of SUMO precursors to complete SUMOylation, but also deSUMOylate substrate proteins, which plays an important role in maintaining the balance between SUMOylation and deSUMOylation [125]. Deregulation of SUMOylation and deSUMOylation leads to cellular dysfunction and is associated to a variety of diseases, including cancer [126]. Therefore, here, we discuss the molecular mechanisms underlying non-histone SUMOylation in cancer progression (Fig. 6).

As one of the most potent modifications of RNA, m6A is involved in many biological processes, especially cancer progression [127, 128]. Human YTH domain family 2 (YTHDF2), one of the main m6A readers, selectively recognizes m6A-RNAs to regulate their degradation [129]. High expression of YTHDF2 together with high level of SUMOylation are linked with cancer progression and predict poor prognosis [130–133]. Hou et al. [134] found that microenvironmental hypoxia in tumors induce SUMOylation of YTHDF2 at the major site of K571. SUMOylation increases the binding affinity of YTHDF2 to m6A-modified RNA, which leads to the degradation of certain RNAs, ultimately promoting tumorigenesis and malignant transformation.

Hexokinase (HK) is the first enzyme of the glycolytic pathway and the rate-limiting enzyme of the glycolytic pathway. Among the known HK isoforms, HK2 is predominant in tumor tissues and can promote tumor progression through its metabolic and non-metabolic functions [135–137]. The oncogenic potential of HK2 is controlled by its cellular localization, a process that relies on the binding of HK2 to the outer membrane of mitochondria. HK2 was found to be SUMOylated at K315 and K492, and SENP1 mediated the deSUMOylation of hexokinase 2 [138]. Notably, SUMO-deficient HK2 binds better to mitochondria and enhances glycolysis in cancer cells. This metabolic reprogramming promotes proliferation and chemoresistance of prostate cancer cells. The SENP1-HK2 axis has the potential to be an entry point to address chemoresistance in prostate cancer. Preclinical studies have initially demonstrated the potential benefits of SENP1 inhibitors in the treatment of cancer [139, 140].

SUMOylation does not act alone, but often regulates various pathological activities of cancer through complex interactions with other PTMs [141]. This crosstalk plays an important role in the occurrence and progression of cancer. A deeper understanding of the crosstalk between SUMOylation and other PTMs may help develop new cancer treatments.

## Conclusion

Epigenetics is a reversible dynamic process that regulates gene expression without altering DNA. PTMs increase the functional diversity of the proteome through the covalent addition of functional groups or proteins, proteolytic cleavage of regulatory subunits, or degradation of whole proteins. The development of mass spectrometry-based techniques for the identification and quantification of protein PTMs has led to an understanding of the critical role of non-histone PTMs in cancer. In this review, we focus on the role of common non-histone PTMs in cancer, such as acetylation, lactylation, ubiquitination, methylation, phosphorylation, and SUMOylation. To be sure, PTMs of non-histone proteins are critical to the occurrence and development of cancer. However, more research on non-histone PTMs is warranted. For instance, considering the heterogeneity and plasticity of the tumor microenvironment, whether non-histone PTMs play the same role in different cancer types and patients? Besides, new and unknown post-translationally modified non-histone substrates and their regulatory mechanisms for cancer progression need to be discovered. We eagerly await more studies to help us understand the mechanism of action of non-histone PTMs in cancer progression and develop better treatment options for cancer patients.
